# Neurovascular and immune factors of vulnerability of substantia nigra dopaminergic neurons in non-human primates

**DOI:** 10.1038/s41531-024-00735-w

**Published:** 2024-06-17

**Authors:** Tiziano Balzano, Natalia López-González del Rey, Noelia Esteban-García, Alejandro Reinares-Sebastián, José A. Pineda-Pardo, Inés Trigo-Damas, José A. Obeso, Javier Blesa

**Affiliations:** 1 HM CINAC (Centro Integral de Neurociencias Abarca Campal), Hospital Universitario HM Puerta del Sur, HM Hospitales, Madrid, Spain; 2 Instituto de Investigación Sanitaria HM Hospitales, Madrid, Spain; 3 Aligning Science Across Parkinson’s (ASAP) Collaborative Research Network, Chevy Chase, MD USA; 4 PhD Program in Neuroscience Autónoma de Madrid University-Cajal Institute, Madrid, Spain; 5 Network Center for Biomedical Research on Neurodegenerative Diseases (CIBERNED), Instituto Carlos III, Madrid, Spain; 6 Facultad HM de Ciencias de la Salud de la Universidad Camilo José Cela, Madrid, Spain

**Keywords:** Parkinson's disease, Neuroimmunology

## Abstract

Dopaminergic neurons in the ventral tier of the substantia nigra pars compacta (SNc) degenerate prominently in Parkinson’s disease (PD), while those in the dorsal tier and ventral tegmental area are relatively spared. The factors determining why these neurons are more vulnerable than others are still unrevealed. Neuroinflammation and immune cell infiltration have been demonstrated to be a key feature of neurodegeneration in PD. However, the link between selective dopaminergic neuron vulnerability, glial and immune cell response, and vascularization and their interactions has not been deciphered. We aimed to investigate the contribution of glial cell activation and immune cell infiltration in the selective vulnerability of ventral dopaminergic neurons within the midbrain in a non-human primate model of PD. Structural characteristics of the vasculature within specific regions of the midbrain were also evaluated. Parkinsonian monkeys exhibited significant microglial and astroglial activation in the whole midbrain, but no major sub-regional differences were observed. Remarkably, the ventral substantia nigra was found to be typically more vascularized compared to other regions. This feature might play some role in making this region more susceptible to immune cell infiltration under pathological conditions, as greater infiltration of both T- and B- lymphocytes was observed in parkinsonian monkeys. Higher vascular density within the ventral region of the SNc may be a relevant factor for differential vulnerability of dopaminergic neurons in the midbrain. The increased infiltration of T- and B- cells in this region, alongside other molecules or toxins, may also contribute to the susceptibility of dopaminergic neurons in PD.

## Introduction

The main neuropathological features of PD is the loss of dopaminergic neurons in the substantia nigra pars compacta (SNc)^1^. Cardinal motor features (akinesia, rigidity, and tremor) generally appear only after 40–60% of dopaminergic neurons are lost, and striatal dopamine (DA) concentration falls below 60–70%^1,2^. However, some dopaminergic midbrain neurons survive even into the late stages of the disease, suggesting differential vulnerability to degeneration. Specifically, dopaminergic neurons in the ventral SNc are highly vulnerable, while dopaminergic neurons in the dorsal SNc and ventral tegmental area (VTA) demonstrate a much lower degree of degeneration^3–6^. To date, the factors underlying differential vulnerability within this defined cytoarchitectural region are still unknown^7^.

Several factors might contribute to selective vulnerability of SNc DA neurons in PD, including oxidative stress, DA toxicity, iron content, autonomous pacemaking, axonal arborization size, lack of calcium-binding proteins such as calbindin-D28K, and gene factors^4,7,8^. One commonality between these factors is that they all suggest that vulnerable neurons are under intense bioenergetic demand^9^. Regulation of tissue energy supply and cellular energy metabolism is essential to maintain healthy cellular and systemic function^10^. Energy requirements are therefore not uniform throughout the brain but instead are increased in localized regions dependent on neuronal activity. Altered transport of molecules (including toxins) between blood and brain, aberrant angiogenesis, vessel regression, and inflammatory responses may contribute to the different neuronal vulnerability in PD^11–14^. The neuroinflammatory scenario of PD includes glial cell activation^15^, immune cell infiltration into the central nervous system (CNS)^16,17^, and increased pro-inflammatory cytokines/chemokines in the brain parenchyma^18–21^. Still, the link between SNc dopaminergic neuron vulnerability, glial and immune cell responses, vascular alterations, and how they contribute to neurodegeneration in PD remains unclear^14^.

Thus, we aimed to investigate the contribution of glial cell activation and immune cell infiltration in selective vulnerability of dopaminergic neurons, as well as to assess structural changes in the vascular system within specific midbrain regions in a well-established non-human primate model of PD.

## Results

### Differential degeneration of dopaminergic neurons in MPTP primates

**Fig. 1:**
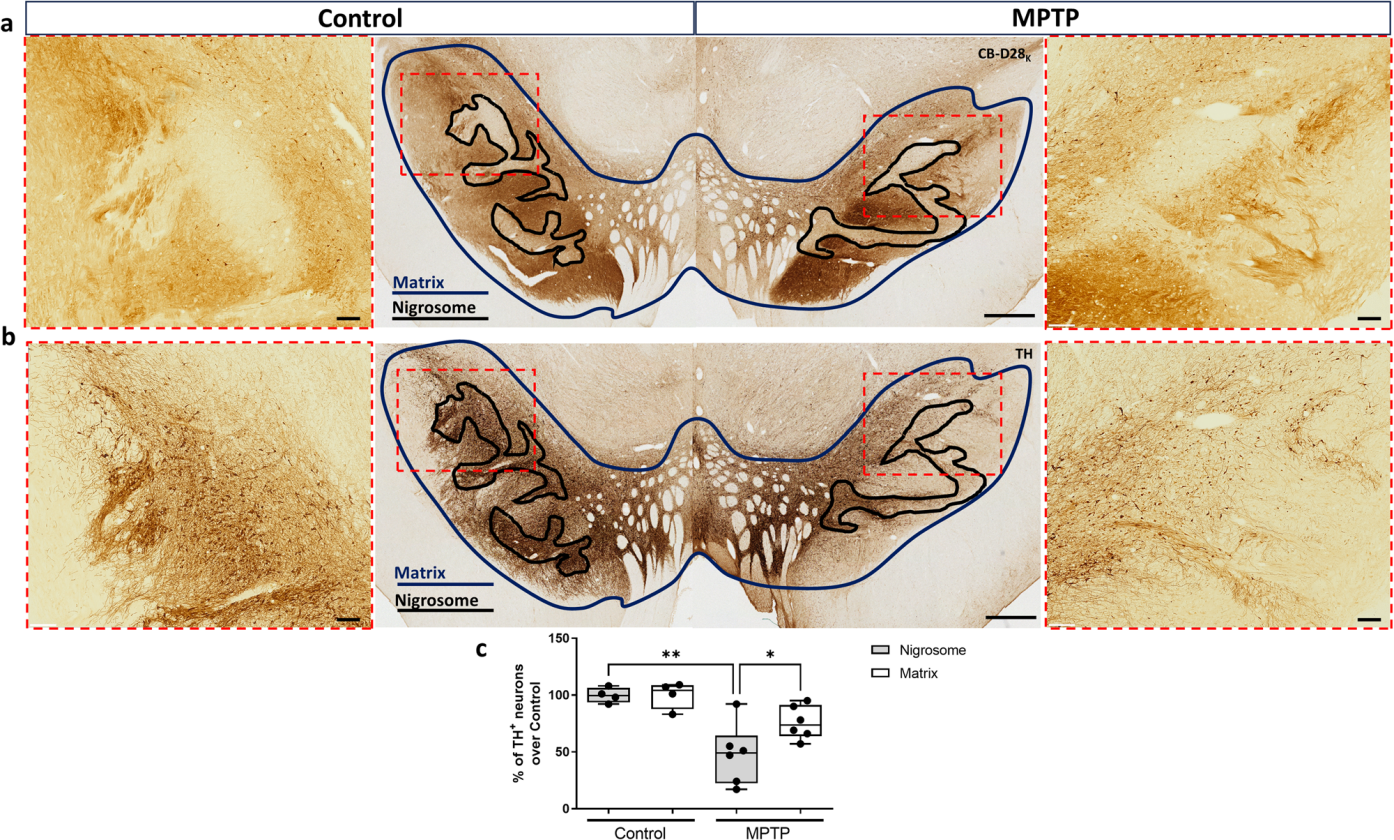
Differential vulnerability of midbrain dopaminergic cells. Calbindin immunostained sections were used as a template to subdivide the dopaminergic midbrain region in the nigrosome (CB-D_28K_-poor zone, outlined in black) and the matrix (CB-D_28K_-rich zone, outlined in blue) (**a**). Outlined regions were then applied on adjacent serial sections immunostained with TH (**b**). Box and whisker plots with median, percentiles, and individual values of 4 control and 6 MPTP monkeys display the number of dopaminergic cells within the nigrosome vs. *the matrix* (**c**). Neuronal loss was higher in the nigrosome than the matrix of MPTP monkeys compared to the same regions in control monkeys. Two-way ANOVA and multiple comparisons were applied to determine which pairs were significantly different. A confidence level of 95% was accepted as significant. *P < 0.05; **P < 0.01. Scale bar of low magnification images = 1 mm; Scale bar of high magnification images = 200 μm.

We first confirmed the higher vulnerability of the ventral dopaminergic neurons including the nigrosome by stereological analysis of TH^+^ neurons within the whole dopaminergic midbrain. Sections immunostained for calbindin were used as a template to subdivide the dopaminergic midbrain region in two regions: the nigrosome (CB-D_28K_-poor zone) and the matrix (CB-D_28K_ rich-zone) as described previously^22^ (Fig. 1a). The outlined regions were then applied on adjacent serial sections immunostained with TH, and the numbers of dopaminergic cells within these two regions were quantified (Fig. 1b). Neuronal loss was higher within the nigrosome of MPTP monkeys, where more than 50% of dopaminergic neurons degenerated in comparison with control monkeys (Fig. 1c). In contrast, the neurons contained in the matrix of MPTP monkeys showed only a 24% loss compared with the control monkeys (Fig. 1c).

### Glial response patterns in MPTP-induced neurodegeneration

**Fig. 2:**
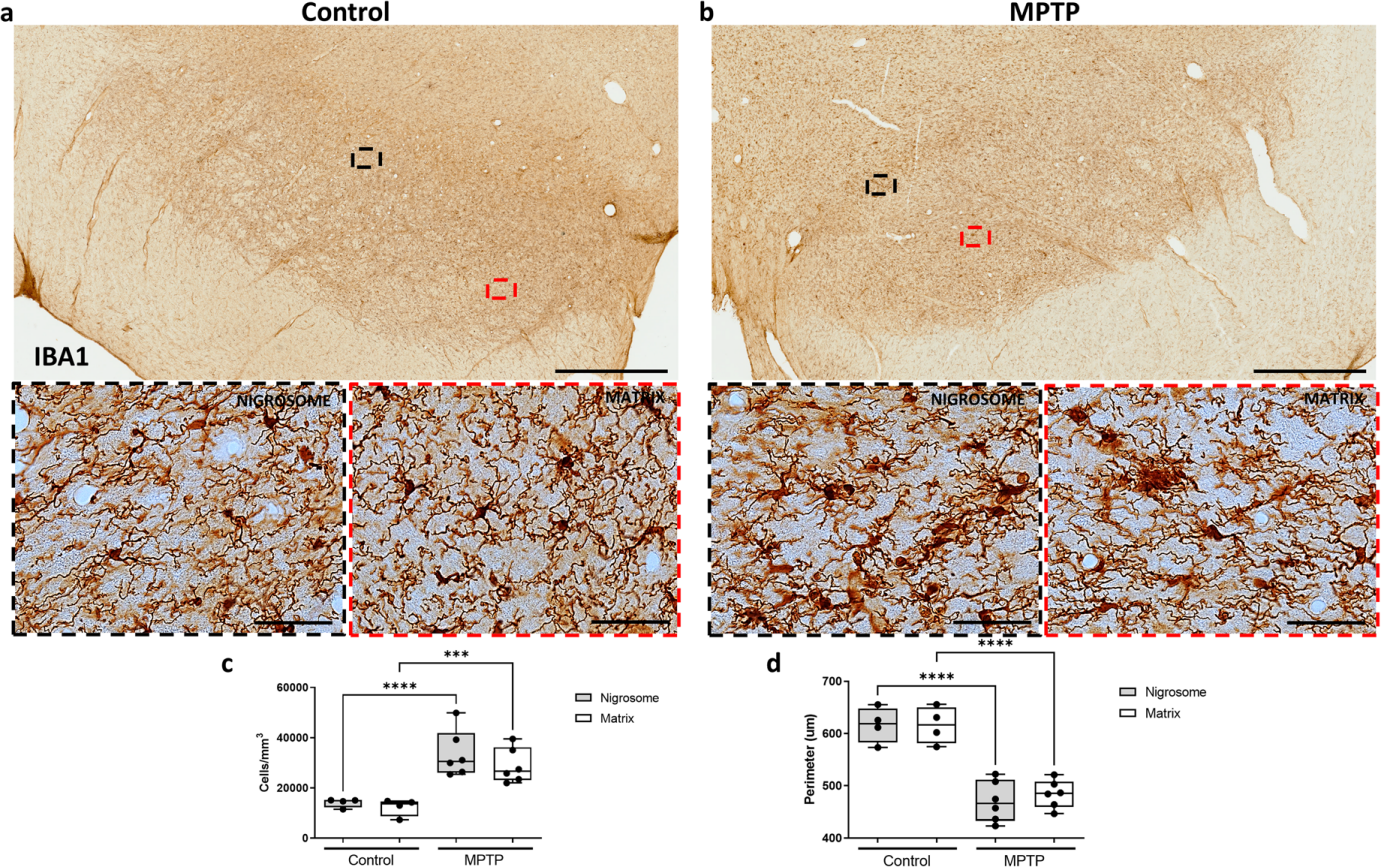
Regional distribution and activation state of microglial cells in the midbrain of control and parkinsonian monkeys. Low and high magnification representative images of two sections from a control (**a**) and an MPTP monkey (**b**) immunostained with IBA1 are shown. Box and whisker plots with median, percentiles, and individual values of 4 control and 6 MPTP monkeys display numerical (**c**) and morphological (**d**) values of microglial cells. MPTP monkeys show massive glial activation in the whole midbrain, but no major inter-regional differences were observed in comparing the nigrosome and matrix. Two-way ANOVA and multiple comparisons were applied to determine which pairs were significantly different. A confidence level of 95% was accepted as significant. ***P < 0.001; ****P < 0.0001. Scale bar of low magnification images = 1 mm; Scale bar of high magnification images = 50 μm.

**Fig. 3:**
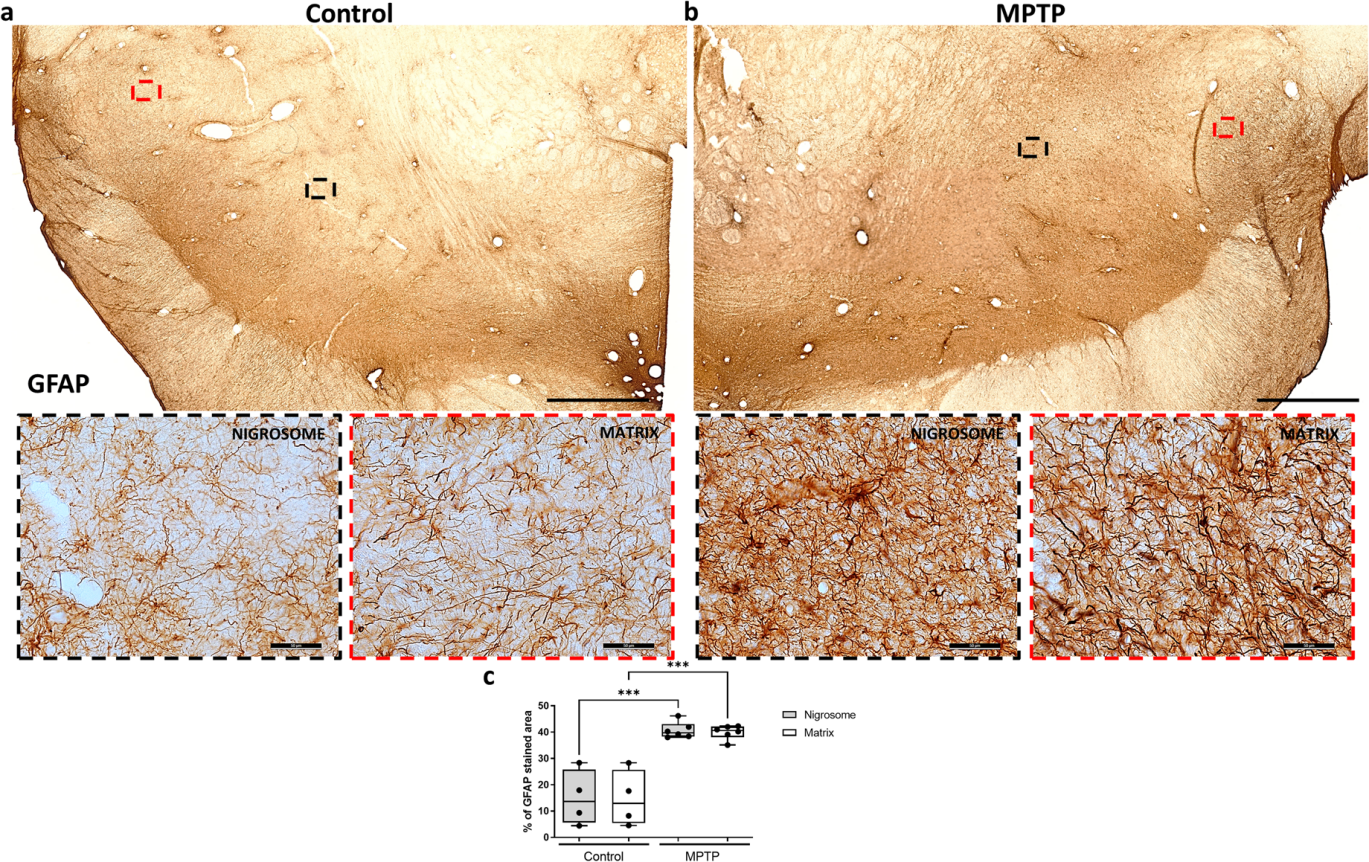
Regional astrocytic response in the midbrain of control and parkinsonian monkeys. Low and high magnification representative images of two sections from a control (**a**) and an MPTP monkey (**b**) immunostained with GFAP are shown. Box and whisker plots with median, percentiles, and individual values of 4 control and 6 MPTP monkeys display the percentage of GFAP stained area in groups and regions (**c**). MPTP monkeys show more GFAP content in the whole midbrain, but no major inter-regional differences were observed in comparing the nigrosome and matrix. Two-way ANOVA and multiple comparisons were applied to determine which pairs were significantly different. A confidence level of 95% was accepted as significant. ***P < 0.001. Scale bar of low magnification images = 1 mm; Scale bar of high magnification images = 50 μm.

Then, the same CB-D_28K_-outlined regions were equally applied on adjacent serial sections immunostained for different markers in each monkey. We first assessed whether glial populations were differently distributed along these regions. No differences were observed in comparing microglial cell numbers (Fig. 2) and GFAP content (Fig. 3) between the nigrosome and matrix of control monkeys, suggesting that glial cells equally populate these regions. An increase in microglial cells (Fig. 2c; *P* < 0.001) and a decreased microglial perimeter (Fig. 2d; *P* < 0.001), reflecting microglial proliferation and activation, were observed both in the nigrosome and the matrix of MPTP monkeys. MPTP-treated monkeys displayed significantly more microglial cells with ameboid morphology (Fig. 2b) in comparison with the control group, presenting fewer cells and a predominant ramified and surveilling morphology (Fig. 2a). Similarly, more GFAP content, reflecting astrogliosis, was observed in both the nigrosome and the matrix of MPTP monkeys compared with the control group (Fig. 3; *P* < 0.001).

### Immune cell infiltration and vascular dynamics

**Fig. 4:**
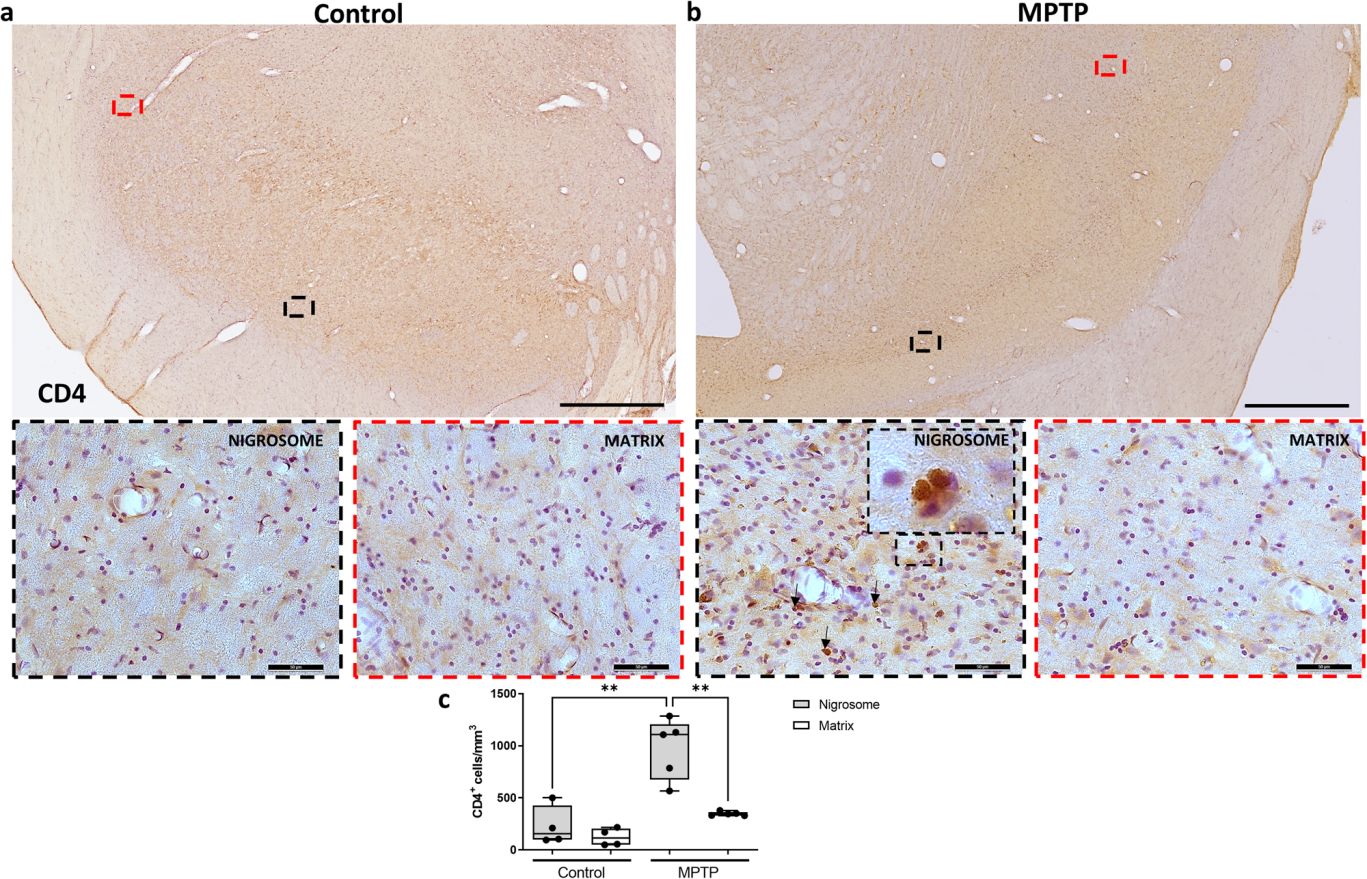
Differential T cell infiltration within the dopaminergic midbrain. Low and high magnification representative images of two sections from a control (**a**) and an MPTP monkey (**b**) immunostained with CD4 are shown. Box and whisker plots with median, percentiles, and individual values of 4 control and 5 MPTP monkeys display the number of infiltrating T cells estimated by stereology (**c**). Increased T cell infiltration was observed in the nigrosome but not in the matrix of MPTP monkeys in comparison with control monkeys. Arrows and insets indicate some examples of infiltrated T cells in the analyzed brain regions. Two-way ANOVA and multiple comparisons were performed to determine which pairs were significantly different. A confidence level of 95% was accepted as significant. **P < 0.01. Scale bar of low magnification images = 1 mm; Scale bar of high magnification images = 50 μm.

**Fig. 5:**
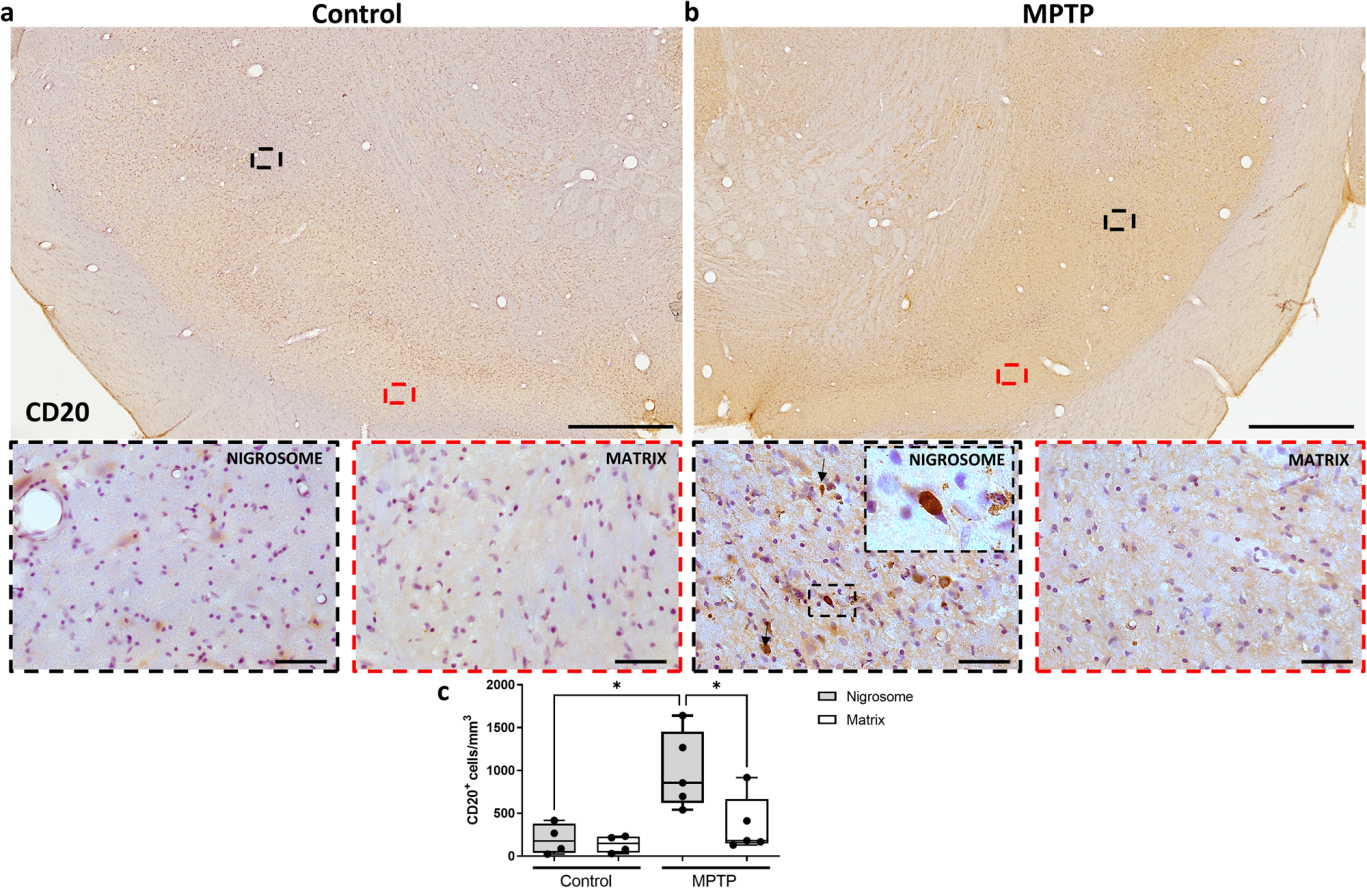
Differential B cell infiltration within the dopaminergic midbrain. Low and high magnification representative images of two sections from a control (**a**) and an MPTP monkey (**b**) immunostained with CD20 are shown. Box and whisker plots with median, percentiles, and individual values of 4 control and 5 MPTP monkeys display the number of infiltrating B cells estimated by stereology (**c**). Increased B cell infiltration was observed in the nigrosome but not in the matrix of MPTP monkeys in comparison with control monkeys. Arrows and insets indicate some examples of infiltrated B cells in the analyzed brain regions. Two-way ANOVA and multiple comparisons were performed to determine which pairs were significantly different. A confidence level of 95% was accepted as significant. *P < 0.05. Scale bar of low magnification images = 1 mm; Scale bar of high magnification images = 50 μm.

Several studies have revealed infiltration of T and B lymphocytes in the substantia nigra of PD patients and PD animal models^16,23,24^. We thus investigated whether this infiltration was region dependent. Stereological analysis of sections immunostained against CD4 (Fig. 4) and CD20 (Fig. 5), markers of T and B lymphocytes, respectively, revealed infiltration of both cell types within the nigrosome of MPTP monkeys (Fig. 4c; *P* < 0.01 and Fig. 5c; *P* < 0.05).

**Fig. 6:**
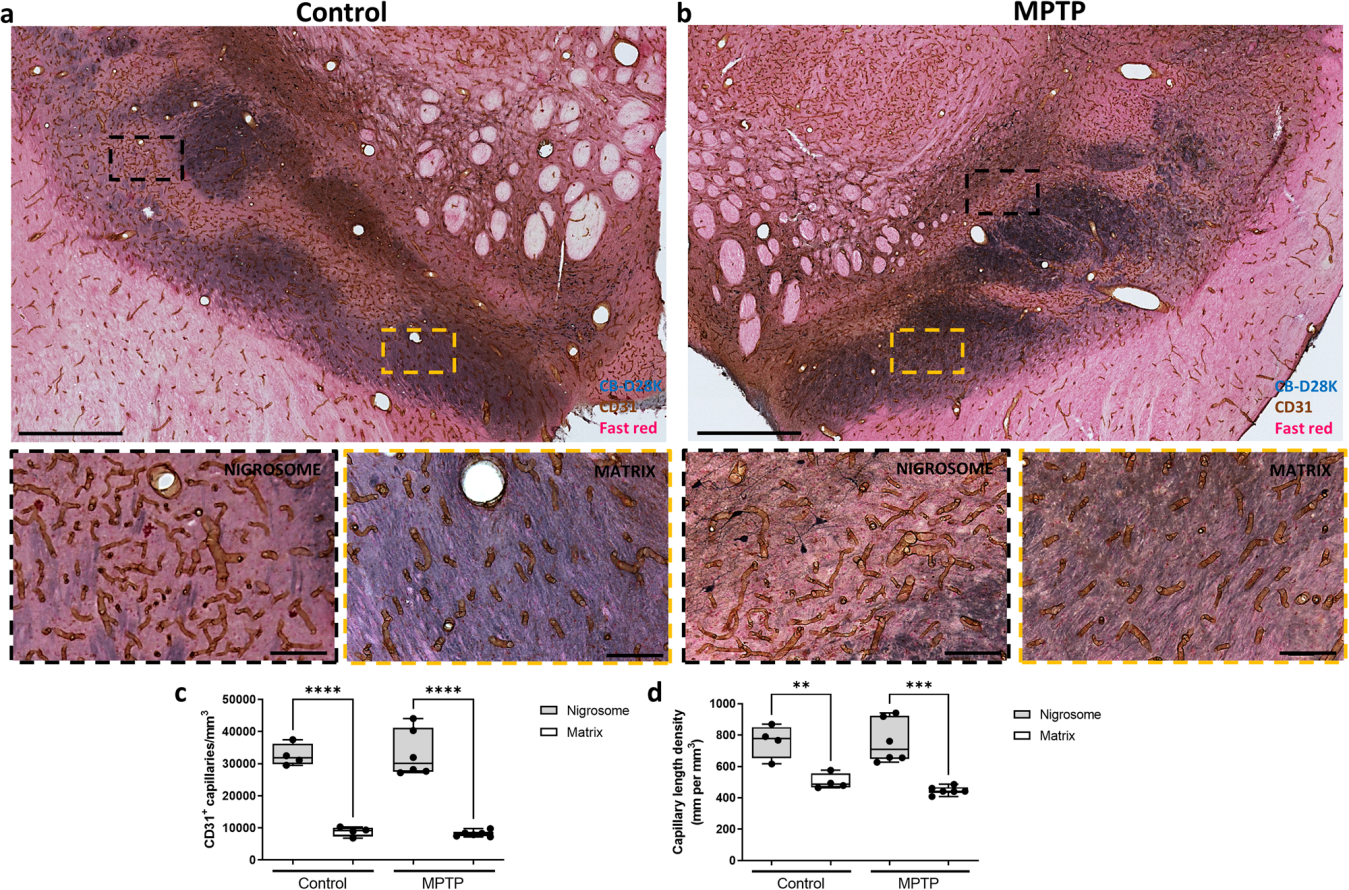
Differential vascular pattern in primate midbrain. Low and high magnification representative images of two sections double-immunostained with CB-D_28K_ (in blue) and CD31 (in brown) (**a**, **b**). Box and whisker plots with median, percentiles, and individual values of 4 control and 6 MPTP monkeys display the density (**c**) and length density (**d**) of capillaries in the nigrosome and matrix quantified by stereology. In control and parkinsonian monkeys, the nigrosome (blue-poor regions) is much more vascularized in comparison with the rest of the dopaminergic midbrain (blue-enriched regions). Two-way ANOVA and multiple comparisons were performed to determine which pairs were significantly different. A confidence level of 95% was accepted as significant. **P < 0.01; ***P < 0.001; ****P < 0.0001. Scale bar of low magnification images = 1 mm; Scale bar of high magnification images = 100 μm.

Previous reports have suggested that structural variations in the vasculature traversing different anatomical regions within the CNS strongly influence where and how CNS immune responses first develop^25^. Thus, we analyzed vascular distribution to investigate how immune cells selectively infiltrate the different dopaminergic midbrain regions. Using the endothelial cell marker CD31 we performed a stereological estimation of density (number of capillaries/mm^3^) and length density (mm of capillaries/mm^3^) of capillaries within the nigrosome and the matrix (Fig. 6). Remarkably, the nigrosome region was more vascularized in control monkeys in comparison with the rest of the dopaminergic midbrain regions (Fig. 6a). Control monkeys showed more capillaries (Fig. 6c; *P* < 0.0001) that were more densely packed (Fig. 6d; *P* < 0.01) within the nigrosome compared to the matrix. MPTP treatment did not alter vascular the distribution (Fig. 6b).

**Fig. 7:**
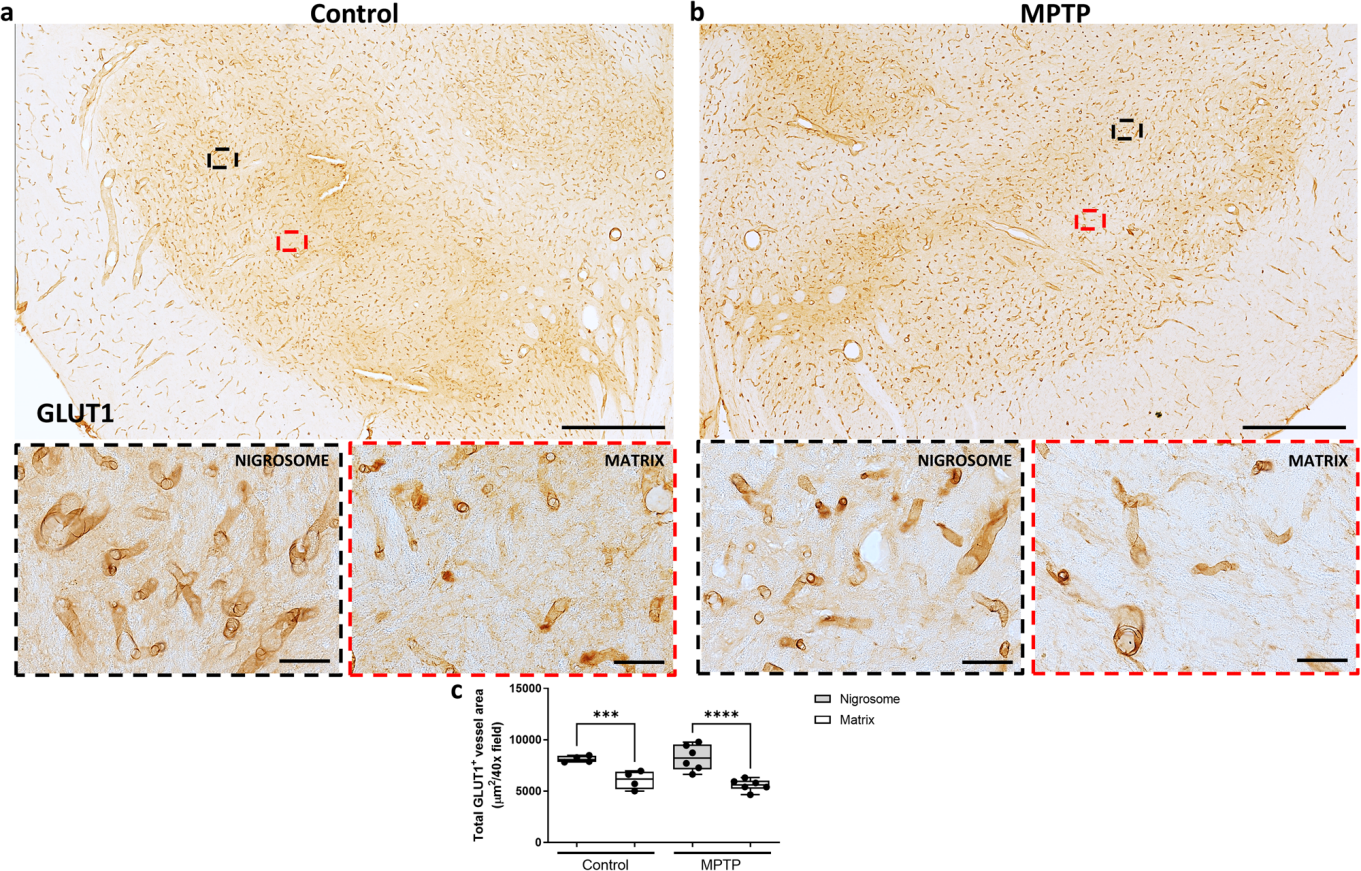
Automated analysis of vascular area in GLUT1 immunostained sections. Low and high magnification representative images of two sections from a control (**a**) and an MPTP monkey (**b**) immunostained with GLUT1 are shown. Box and whisker plots with median, percentiles, and individual values of 4 control and 6 MPTP monkeys display the total GLUT1^+^ vessel area per field by automated quantification by ImageJ (**c**), being higher in the nigrosome than in the rest of the dopaminergic midbrain. Two-way ANOVA and multiple comparisons were performed to determine which pairs were significantly different. A confidence level of 95% was accepted as significant. ***P < 0.001; ****P < 0.0001. Scale bar of low magnification images = 1 mm; Scale bar of high magnification images = 50 μm.

To further confirm these findings, we analyzed the vascular distribution using another vascular marker (GLUT1) and an alternative quantitative method (automated quantification). Automated analysis of GLUT1 immunostained sections confirmed the previous results, showing more capillaries in the nigrosome than in the matrix of both groups (Fig. 7c; *P* < 0.0001). No major difference was found in a comparison of control vs MPTP monkeys (Fig. 7a, b).

## Discussion

We show here that the ventral tier of the SNc, the nigrosome, is normally more densely vascularized than the rest of the midbrain. This unique feature could make this region particularly sensitive to stressors of any sort and also be a privileged route of entry for toxins and immune cells contributing to PD pathology and vulnerability of the ventral SNc. Surprisingly, the role of increased vascularization of the substantia nigra as a possible contributor to selective vulnerability has only been taken into consideration in isolated studies in monkeys^26,27^ and PD patients^28^. The fact that the nigrosome is highly vascularized is not totally unexpected and builds on the distinct features of the SNc ventral region. In fact, the data presented here, together with other studies in non-human primates and human post-mortem brain samples, indicate that the nigrosome is significantly more densely cell populated compared to the rest of the midbrain^3,22,29–31^. Moreover, the ventral SNc is known to be a zone of high energy demand, making it particularly bioenergetically compromised in the human brain^32–35^. Thus, the greater vascularization within the nigrosome may be a normal feature in accordance with its metabolism but could allow the entrance into the brain parenchyma of deleterious factors.

Indeed, we observed preferential entrance of lymphocytes into the nigrosome. We can speculate that the greater vascularization in this region may have acted as a privileged route of entry for these cells. Admittedly, our neurotoxic model and approach does not allow further elucidation of a possible specific pathogenic role of lymphocytes. To what extent this distinctive infiltration could play a role in the different vulnerability of midbrain neurons or is just a consequence of the higher dopaminergic cell death needs to be more definitively addressed. Nevertheless, a consistent body of literature suggests a pivotal role of infiltrating immune cells within the SNc in the neurodegenerative process of PD^36^. McGeer and colleagues described a substantial presence of CD8 + T cells in the post-mortem brain in a PD patient^23^. Stronger quantitative evidence from a larger number of subjects revealed infiltration of CD4+ and CD8 + T cells specifically in the substantia nigra of 14 PD patients^16^. More recently, it has been suggested that immune cell invasion into the substantia nigra may even precede the dopaminergic neuronal loss in this region^17^. Also, mice overexpressing α -synuclein in the midbrain developed upregulation of the major histocompatibility complex II (MHCII) protein on microglia, macrophages, and monocytes, accompanied by infiltration of CD4 and CD8 T cells into the SNc^37^. More evidence along this line was obtained in several neurotoxin models of PD induced by MPTP^38,39^ and 6-hydroxydopamine^40^. Certainly, reducing the activation or entrance of T-cells with immunosuppressive drugs or reducing the deleterious effects of peripheral immune activity led to reduced deposits of pathogenic forms of α-synuclein, low-grade neuroinflammation, and reduced neurodegeneration^37,41–45^.

While T cells have been extensively studied in the brains of post-mortem PD patients and animal models, research into the role of B cells to date has been limited. In steady-state conditions, B cells enter all areas of brain parenchyma in low numbers^46^. However, B cells can increase in number and/or effector function under pathological situations (including aging and neurodegenerative diseases)^47^. A possible role of humoral immunity (B cells) in the pathogenesis of PD was first described in a postmortem study with idiopathic and genetic PD. This study found the presence of immunoglobulin G (IgG) binding to dopaminergic neurons and Lewy bodies in the substantia nigra, highlighting a role for antibody-dependent B cell-mediated cytotoxicity in PD^48^. These results were later supported by investigations in PD animal models, unveiling long-term infiltration of B cells in the midbrain of rodents^49–51^ and non-human primates^52^. Yet, further studies to identify the underlying mechanisms mediating this infiltration, considering different sub-regions, and the role of the blood-brain barrier in this scenario are needed.

Finally, we have also investigated the role of glial cells in the selective vulnerability of dopaminergic neurons. Microglia and astrocytes were equally distributed in the entire midbrain in normal monkeys. This result agrees with a previous report that showed no sub-regional differences in glial cell activation state or number in the midbrain of normal young or adult rhesus macaques^53^. As expected, MPTP monkeys displayed increased astrogliosis and microglial proliferation and activation in the entire dopaminergic midbrain, but no sub-regional differences were observed. However, it has been well established that microglia and astrocytes, as part of the neurovascular unit, can exert destructive or reparative functions, responding differently depending upon local extracellular and intracellular signals^54–57^. Thus, it is possible that the greater vascularization and immune response found in the nigrosome, together with other factors already known (neurotoxins or α-synuclein among others), could synergistically promote a pro-inflammatory phenotype and contribute to the neurodegenerative processes in PD.

The results presented here suggest a possible role of vascular elements in the vulnerability of dopaminergic neurons within the substantia nigra in PD. The hyper-vascularization of the nigrosome may serve as a privileged mechanism and route of entry for toxins, immune cells, and other substances under pathological situations. The observed inflammation and infiltration of immune cells are probable outcomes of the loss of dopaminergic cells in this monkey model. Nevertheless, our findings mark the first instance of proposing how specific mechanisms, such as neuroinflammation and immune cell infiltration, may exert a disproportionate influence on this region, likely attributable to its distinctive vascular cytoarchitecture.

## Methods

### Animals and MPTP administration

Ten male macaque monkeys (*Macaca fascicularis*), weighing 5.5–12 kg, aged 5–10 years, and sourced from R.C. Hartelust BV (Tilburg, The Netherlands), were used in this study. The animals were housed in an animal room under standard conditions and treated in accordance with European and Spanish guidelines (86/609/EEC and 2003/65/EC European Council Directives and the Spanish Government). The experimental protocol was approved by the Ethical Committee for Research of the Fundación de Investigación HM Hospitales and of Comunidad de Madrid. Water and fresh fruit were available *ad libitum*. Qualified health care personnel oversaw the monkeys’ welfare throughout the studies.

Six monkeys were treated with 1-methyl-4-phenyl-1,2,3,6-tetrahydropyridine (MPTP, Sigma) by systemic administration (i.v.) under light anesthesia (ketamine 10 mg/kg; i.m.) using a dose regimen of 0.5 mg/kg every 2 weeks to obtain partial, slowly progressive degeneration of the nigrostriatal dopaminergic system as described before^29,58–62^. As each animal has a different susceptibility to MPTP, the number of injections varied depending on the systemic response to the toxin and the motor score reached. Motor status was assessed using the validated Kurlan motor scale^63^. Four animals showed evident parkinsonism after either the first or the second MPTP injections, whereas another two animals showed no apparent parkinsonian features after 3 MPTP injections and did not receive any further doses. The mean motor scale scores in all MPTP monkeys were 10.5 ± 3.5 (after MPTP injections) and 6.5 ± 4.1 (before sacrifice). Four monkeys were used as controls. No animals received l-DOPA or dopaminergic agonizts during the study. Animals were sacrificed from 2 to 14 weeks after the last MPTP injections. In this study, all MPTP monkeys exhibited minimal group variations across all analyzed parameters (inflammatory, immune, and vascular parameters). Additionally, no significant correlation between these parameters and motor scale was observed (data not shown). Therefore, we grouped all MPTP monkeys to increase the statistical power of each analysis.

### Fixation and tissue processing

Monkeys were anesthetized deeply with sodium pentobarbital (10 mg/kg/i.p.) and perfused through the ascending aorta with saline, followed by 4% paraformaldehyde in phosphate buffer (PB) and a series of PB sucrose solutions (5–10–20%). After perfusion, brains were stereotaxically blocked in the coronal plane and sectioned on a sliding microtome at 40 μm to produce 10 matched series.

### Immunohistochemistry

Sections were washed with Tris buffer (TB) and treated with citrate buffer (pH 6) for 30 min at 37 °C for antigen retrieval. Inhibition of endogenous peroxidase activity was performed using a mixture of 10% methanol and 3% concentrated H_2_O_2_ for 20 min. Normal serum was applied for 3 h to block non-specific binding sites. Sections were immunostained at 4 °C for a duration of 72 h using primary antibodies. Sections were washed with Tris-buffered saline (TBS) and transferred for 2 h to a solution containing the corresponding secondary biotinylated antibody. Next, sections were incubated for 45 min with the avidin-biotin-peroxidase complex (ABC Vectastain, Vector Laboratories). Immunohistochemical reactions were visualized by incubating the sections with 0.05% 3.3′-diaminobenzidine (DAB; Sigma) and 0.003% H_2_0_2_.

For double-labeling immunohistochemistry, after DAB staining, sections were washed with TBS at 95 °C for 5 min, and the immunohistochemical procedure was repeated using the next primary and secondary antibodies. Immunohistochemical reactions were visualized by incubating the sections with a blue chromogen (Vector peroxidase substrate kit, Vector Laboratories). Sections were then counterstained with hematoxylin or nuclear fast red for 5 min, dehydrated through ascending series of ethanol and cleared in 2 changes of xylene before being mounted in DPX and applying glass coverslips. The omission of the primary antibody resulted in non-staining (images not shown). All cases (animals) for each individual stain were batch processed under identical parameters.

A complete list of antibodies and reagents, together with incubation concentrations and commercial sources is provided in Table 1.

**Table 1:** Table of primary, and secondary antibodies and reagents used for immunohistochemical analysis

Antibodies and reagents	Target	Dilution	Source and reference	RRID
Mouse anti CB-D_28K_	Calbindin-D_28K_	1:7000	Swant (CB300)	AB_10000347
Mouse anti TH	DA neurons	1:1000	Millipore (MAB5280)	AB_2201526
Rabbit anti IBA1	Microglia	1:1000	Wako (019-19741)	AB_839504
Mouse anti GFAP	Astrocytes	1:2000	Abcam (Ab4648)	AB_449329
Mouse anti CD4	T cells	1:200	Novus Bio (NBP2-46149)	N.A.
Mouse anti CD20	B cells	1:200	Novus Bio NBP2-45454	N.A.
Mouse anti CD31	Endothelial cells	1:100	Dako (M0823)	AB_2114471
Rabbit anti GLUT1	Endothelial cells	1:250	Abcam (Ab14683)	AB_301408
Anti-Mouse IgG (H + L), made in horse	Primary antibody	1:400	Vector laboratories (BA-2000)	AB_2313581
Anti-Rabbit IgG, biotin-SP conjugate, made in goat	Primary antibody	1:400	Chemicon (AP132B)	AB_11212148
VECTASTAIN ELITE ABC-HRP Kit	Biotinylated Antibodies	Manifacturer’s specification	Vector Labs (PK-6100)	AB_2336819
Vector SG Peroxidase substrate	HRP substrate reaction	Manifacturer’s specification	Vector Labs (SK-4700)	AB_2314425
DAB	HRP substrate reaction	0.05%	Sigma-Aldrich (D5637)	N.A.
Mayer’s hematoxylin	Nuclei	Ready to use	Sigma-Aldrich (MHS32)	N.A
Nuclear Fast Red	Nuclei	Ready to use	Sigma-Aldrich (112084/3)	N.A.

### Regional segmentation of midbrain by CB-D_28K_ immunostaining and immunohistochemical analysis

The dopaminergic subgroup boundaries in monkeys and humans are routinely distinguished according to immunoreactivity for TH immunoreactivity and calbindin (calcium-binding protein D_28k_, CB-D_28K_) (Fig. 1). The ventral tier of the SNc (nigrosome) is uniquely identified by its marked absence of CB-D_28K_ immunoreactivity, in both the neuropil and soma of dopaminergic neurons^3,6,22,30,64–71^. We then grouped the CB-D_28K_-rich zones including the dorsal tier of the SNc, VTA, and the substantia nigra reticulata (SNr) as the matrix^22^ (Fig. 1).

The resulting ROIs were then applied on adjacent serial sections immunostained with the other markers to evaluate the role of neuroinflammation, immune cell infiltration, and vasculature in the selective vulnerability of dopaminergic neurons within the different regions.

#### Stereological analysis

Stereological estimation was carried out by using a computer-assisted image analysis system consisting of a microscope (Olympus BX3) equipped with a computer-controlled motorized stage, a camera, and the Stereo Investigator software (Stereo Investigator 2017, MicroBrightField). Four sections regularly spaced at intervals of 1200 μm with the optical fractionator method covering the entire rostro-caudal axis of the SNc were used. All parameters (counting objective, counting frame, and sampling grid) used for the stereological analysis are summarized in Table 2. After the counting was finished, the total number of cells or capillaries was automatically calculated by the software using the formula described by West^72^, and results were expressed as cells or capillaries per mm^3^.

**Table 2:** Summary of all parameters (stereological method, microscope objective, counting frame or sphere radius, and sampling grid) used for the stereological estimation of number or density of dopaminergic cells, microglial cells, T and B lymphocytes and capillaries

Marker	Unit	Method	Microscope objective	Counting frame/radius	Sampling grid
TH	DA cells	Optical Fractionator	40X	150 × 120 μm	350 × 350 μm
IBA1	Microglial cells	Optical Fractionator	40X	100 × 100 μm	250 × 250 μm
CD4	T cells	Optical Fractionator	40X	200 × 200 μm	250 × 250 μm
CD20	B cells	Optical Fractionator	40X	200 × 200 μm	250 × 250 μm
CD31	Capillaries	Optical Fractionator	20X	200 × 200 μm	1500 × 1500 μm
CD31	Capillaries	Spaceballs	20X	20 μm	350 × 350 μm

Capillary length density was also quantified by using the Spaceballs probe as in Wälchli et al.^73^. For this analysis four sections regularly spaced at intervals of 1200 μm were used, and spheres with a radius of 20 μm in a sampling grid of 350 × 350 μm were applied (Table 2). All parameters were set to reach error coefficients below 0.10 (Gundersen, m = 1) as in Gundersen et al.^74^. Error coefficients of the estimates were calculated for each marker.

#### Immunohistochemical quantification of glial cell activation

Four sections per animal, regularly spaced at intervals of 1200 μm were used. All images were photographically recorded under the optical microscope (Olympus BX3). For the analysis of microglial activation, 20 40x-fields per section were used and the perimeter of microglial cells was quantified as in^75^ using the image analysis software (Image-Pro Plus 6.0.0.26, Media Cybernetics, Inc. Rockville). Briefly, all images were first converted from pixel to micrometers using the microscope calibration scale bar. Then, applying an intensity threshold (histogram-based manual intensity range selection) and size filter (area filter range), only microglial cells were selected. In our case, the intensity threshold was 0–180, and the size filter was 150-infinity μm^2^. The average perimeter of all microglia in the selected area was measured, and the results were expressed in micrometers, being lower in the ameboid activated microglia.

The analysis of astrocyte activation was performed using ImageJ 1.52a software. Twenty 40x-fields per section were used. The GFAP^+^ cells were selected by using the *Phansalkar* method from the *Auto Local Threshold* function, and no filter size was applied. Results were expressed as the percentage of GFAP covered area, being higher in case of astrogliosis.

### Automated quantification of vascular area

Four sections per animal, regularly spaced at intervals of 1200 μm, were used. Automated analysis of the vascular area within the midbrain was performed on GLUT1 stained sections. All the images (20 40x-fields per sections) were photographically recorded under the optical microscope (Olympus BX3) and quantified with ImageJ 1.52a software. First, all images were converted from pixel to micrometers using the microscope calibration scale bar. A split channel function was used, and green channel images were selected. Then, applying an intensity threshold (“*default*” method from “*auto threshold*” function) and size filter (10000-infinity pixels), only capillaries were selected. The total vessel area was measured, and results were expressed in μm^2^/40x field.

### Statistical analysis

All statistical analyses were performed using GraphPad Prism software. For all immunohistochemistry experiments, two-way ANOVA and multiple comparisons were performed to determine which pairs were significantly different. Statistical significance was defined as *P* < 0.05. An asterisk “*” indicates a significant difference between two regions of the same experimental group or between the same regions of two different experimental groups. All results were graphically presented using box and whisker plots depicting the median, percentiles, and individual values.

## Data Availability

The datasets generated during the current study are available in the *Zenodo* repository (https://zenodo.org/records/10977929).
